# “We are not gays… don’t tell me those things”: engaging ‘hidden’ men who have sex with men and transgender women in HIV prevention in Myanmar

**DOI:** 10.1186/s12889-018-6351-3

**Published:** 2019-01-14

**Authors:** Vanessa Veronese, Emily Clouse, Andrea L. Wirtz, Kaung Htet Thu, Soe Naing, Stefan D. Baral, Mark Stoové, Chris Beyrer

**Affiliations:** 1 Disease Elimination Program, Burnet Institute, Melbourne, Australia; 2 Center for Public Health and Human Rights, Department of Epidemiology, Johns Hopkins School of Public Health, Baltimore, USA; 3 Alliance Myanmar, Yangon, Myanmar; 4 Department of Epidemiology and Preventive Medicine, Monash University, Melbourne, Australia

**Keywords:** Men who have sex with men, Myanmar, HIV testing, Stigma and discrimination, Sexual orientation concealment

## Abstract

**Background:**

In Myanmar, HIV is concentrated among key populations, yet less than half of the estimated 250,000 men who have sex with men (MSM) and transgender women (TW) report recent HIV testing. As many as 50% of MSM and TW may conceal their same-sex preferences and behaviors, yet little is known about the barriers faced by those who are locally regarded as ‘hidden’ – that is, MSM who do not disclose same-sex preferences and/or identify as gay. This study explored specific barriers to accessing HIV testing and other prevention services among ‘hidden’ MSM to inform appropriate models of service delivery.

**Methods:**

In-depth interviews with MSM (*n* = 12) and TW (*n* = 13) and focus group discussions (FGD) with MSM and TW community members, leaders and key informants (*n* = 35) were undertaken in Yangon during June – September 2015. Participants were recruited by word-of-mouth by trained peer data collectors. Responses to questions from semi-structured guides were transcribed and coded using Atlas Ti. Codes were based on key domains in the guides and applied to transcripts to identify and analyze emerging themes.

**Results:**

Fear of stigma and discrimination and the need to meet gender expectations were key reasons for non-disclosure of same-sex preferences and behaviors; this typically manifested as avoidance of other MSM and settings in which sexual identity might be implicated. These concerns influenced preference and interaction with HIV services, with many avoiding MSM-specific services or eschewing HIV testing services entirely. The difficulties of engaging hidden MSM in HIV prevention was strongly corroborated by service providers.

**Conclusion:**

Hidden MSM face multiple barriers to HIV testing and prevention. Strategies cognizant of concerns for anonymity and privacy, such as One-Stop Shop services and online-based health promotion, can discretely provide services appropriate for hidden MSM. Enhanced capacity of peer-service providers and mainstream health staff to identify and respond to the psychosocial challenges reported by hidden MSM in this study may further encourage service engagement. Overarching strategies to strengthen the enabling environment, such as legal reform and LGBTI community mobilisation, can lessen stigma and discrimination and increase hidden MSM’s comfort and willingness to discuss same-sex behavior and access appropriate services.

## Background

Against a background of low and decreasing global HIV prevalence, men who have sex with men (MSM) and transgender women (TW) continue to be disproportionately affected by HIV [1, 2]. In Asia, HIV prevalence greater than 10% has been observed in some MSM and TW communities [1, 3–5], while emergent epidemics in countries as diverse as Thailand [6], India [7] and the Philippines [8] have been recently noted. This ongoing vulnerability of MSM and TW to HIV acquisition may suggest limitations in both the scale and effectiveness of current HIV prevention responses in the region. This includes Myanmar, where the estimated HIV prevalence among MSM and TW is over 11% nationally, and the concentrations reported in major urban cities comprise some of the highest rates seen across Asia [4]. Despite known risks, less than half of all MSM and TW in Myanmar reported receiving an HIV test in the past 12 months and coverage of HIV prevention programs remains suboptimal [4].

Global elimination targets suggest that ending AIDS by 2030 will require 90% of people living with HIV knowing their status [9]. As well as facilitating early initiation of HIV treatment, HIV testing provides an important entry point to bio-medical HIV prevention strategies such as PrEP [10]. Controlling the HIV epidemic requires regular engagement of MSM and TW at risk of or living with HIV in prevention, treatment and care; this includes those that do not or are unwilling to identify as MSM or TW and may be out of reach of specific HIV programs targeting these groups. In Myanmar, as in other Asian countries, a number of indigenous and diverse gender and sexual identities exist among same-sex attracted men. Unlike typical Western characterizations that utilize separate categories to define sexual and gender identities, one set of labels are often used across Asia to characterize both sexual and gender identities, such as *Kothis* in India and *Bóng lộ* in Vietnam [11, 12]. These labels typically encompass sexual positioning, power dynamics, and gender expression and are reflective of one’s willingness to disclose their sexual preferences, behaviors and gender identity [4, 13].

There are three main local terms used to classify sexual and gender identity among MSM and TW in Myanmar. *Apwint* are commonly regarded as transgender women, and locally understood as individuals with male assignation at birth but who openly identify as female and attracted to men. Conversely, *Apone* are males that typically have a masculine presentation, are sexually oriented towards other men yet conceal their sexual preferences in most social spheres or circumstances and are often locally referred to as ‘hidden, or ‘hider’ for their presentation as ‘men’ in public and certain social environments. *Apwint* and *Apone* are thought to share the same ‘feminine’ inner self, but differ in their outward gender expression [14]. Lastly, *Thange* are males with masculine presentations who also engage in sex with other men, yet do so sporadically or incidentally while also maintaining a heterosexual identity and relationships with women [14–18]. There are an estimated 250, 000 MSM and TW in Myanmar (estimates not disaggregated by gender identity) and as many as 50% are thought to conceal same-sex preferences and behaviors [18].

In Myanmar, sexual and gender identity has been associated with differences in sexual positioning, age of sexual debut, experiences of forced sex, condom use at last sex and access to HIV-related services among MSM and TW and is therefore an important consideration for understanding HIV risk and vulnerability [4, 17, 19, 20]. While in the scientific literature, the term MSM is utilized to reflect behavioral characteristics among male-identified individuals with the intention of respecting individual sexual identity, in Myanmar TW do not consider the term MSM to be restricted only to male-identified individuals and participate in and lead many MSM programs and activities [14]. Additionally, much of the research conducted with MSM and TW is not representative or inclusive of the diversity among sexual minorities in Myanmar. In one of the only published studies on characteristics and risk behaviors among MSM and TW, the majority of participants reported their occupation as beautician or *Nat Kadaw* (spiritual dancer), roles typically assumed by TW [15, 16]. While some literature is beginning to emerge that disaggregates findings by sexual and gender identity [21], very little specifically addresses subgroups of MSM in Myanmar who are locally regarded as ‘hidden’: those that do not disclose same-sex preference or behaviour or identify as gay. Performance of gender and expression of sexuality among same-sex attracted men in Myanmar is further complicated by the legal environment in which they exist; under Myanmar law, homosexual sex remains criminalised and sexual minorities continue to report ongoing discrimination and harassment [22].

Myanmar’s National Strategic Plan for HIV and AIDS 2016–2020 includes a target of reaching 90% of MSM and TW with combination HIV prevention services and prioritizes the engagement of non-disclosing, or ‘hidden’ MSM [4]. A growing body of evidence from the region highlights the ways in which non-disclosure of sexual identity affects HIV service utilization [11, 23, 24], yet much remains unknown about the barriers faced by hidden MSM in Myanmar in accessing HIV services, hindering the development of culturally-relevant and responsive HIV prevention approaches. This formative research was undertaken to better understand the challenges and experiences of MSM and TW in accessing HIV testing, prevention and care and was conducted as part of a broader implementation science study to improve MSM and TW’s access and retention in the HIV Care Cascade in Myanmar [25, 26]. This paper presents a secondary analysis that explores these issues as they specifically relate to hidden MSM and seeks to further our understanding of the characteristics of this group and the barriers and challenges to engaging with HIV prevention services. We consider the perspectives of hidden MSM, the broader MSM and TW community and the service providers and community leaders who seek to engage hidden MSM in HIV prevention. We have included the perspectives of TW in relation to the experiences of hidden MSM in light of the fluidity of sexual and gender identity in Myanmar (i.e., individuals may move between MSM and transgender identities, often depending of social contexts), the common experiences of TW and MSM (e.g., stigma and marginalisation) and the social and physical spaces TW and MSM share in Myanmar.

## Methods

Data were originally collected as a formative study to inform a trial to develop and test novel approaches to HIV testing and engagement in HIV care among MSM and TW in Myanmar led by Johns Hopkins University [25, 26]. In-depth interviews (IDI) were conducted with MSM and TW community members to explore personal information and perspectives related to the following domains: characteristics of MSM and TW; social context for MSM and TW including experiences of stigma; availability and accessibility of HIV testing services; knowledge and perceptions on HIV self-testing; preferences for HIV testing, treatment and care models, and; issues related to operational implementation. Eligibility criteria for IDIs was defined as: biologically male, aged 18 years and above, reported anal sex with a male in the past 12 months, resident of greater Yangon and spoke Myanmar. Focus group discussions (FGDs) were also conducted with MSM and TW community leaders and service providers to explore HIV prevention, treatment and care availability and delivery, and participant experiences related to service provision. Those who reportedly engaged in providing services to MSM and TW for 1 year or more, were aged 18 years and over, and spoke Myanmar were eligible for FGDs.

IDIs and FGDs were recorded, transcribed and translated into English. English transcripts were checked for accuracy by local study staff and subsequently entered into Atlas. Ti (Cincom Systems, Berlin). Data from IDIs and FGDs were analyzed collectively using open interpretive coding. Research team members (AW, EC, VV) independently coded transcripts after establishing consistency in the use of codes across team members. Codes were developed based on key domains in the interview guides and openly applied to transcripts to identify and analyze emerging themes. Regular meetings were held to ensure ongoing consistency of coding. A community validation workshop was held in September 2016 in Yangon during which the preliminary findings and analysis were shared with community interviewers and facilitators from the study to verify and validate authors’ interpretations.

A secondary analysis of this data was undertaken to better understand the characteristics of hidden MSM in Myanmar and the barriers and challenges to engaging them in HIV prevention. Specific domains used for this paper include: experiences related to the MSM and TW community; knowledge, preference and perceptions related to HIV prevention, treatment and care services; experiences of stigma and discrimination and access to HIV-related services.

While government literature utilizes the term ‘non-disclosing’ MSM to characterize MSM who do not openly identify their sexual or gender identity, in this paper we utilise the term ‘hidden’ to reflect local parlance and ensure consistency with participant data. The term ‘hidden MSM’ is therefore applied to participants who were purposively recruited to the ‘hidden MSM’ FGD or who identified as hidden or *Apone*, or those who described concealing behaviors in their interview narratives. Our reference to local identity labels throughout this paper is done with fidelity to the way in which they were utilized by participants throughout the study to convey local understanding and interpretation of  these labels and identities.

We compare and contrast the barriers and experiences of hidden MSM to other MSM and TW and to the perspectives of the services providers and community leaders who seek to engage them in services, in order to highlight the implications for service provision. The analysis of data in this light resulted in the identification of three broad themes which have been used to organize and discuss our data below. Overall participant characteristics are described in Table 1.

**Table 1:** Characteristics of in-depth interviews and focus group discussion participants held in Yangon

In-depth interview participants	n (*n* = 25)	%
Median age, years (range)	23	(18–42)
18–25 years	16	64
26–35 years	5	20
36 years and above	2	8
Sexual identity		
TW	13	52%
MSM	12	48%
Current relationship status		
Single	10	40
Casual partner(s)	4	16
Regular male partner (s)	7	28
Sex worker	3	12
HIV status		
HIV positive	8	32
HIV negative	16	64
HIV unknown/not disclosed	1	4
Focus group discussion participants (n)	Characteristics	
FGD 1: MSM and TW Community leaders (n = 9)	MSM and TW Outreach workers, peer educators and supervisors, volunteers and community workers; both HIV positive and negative participants	
FGD 2: Hidden MSM (*n* = 6)	Hidden MSM, both HIV positive and negative participants, low and middle income status	
FGD 3: Service providers (*n* = 8)	MSM Outreach worker, Peer educators and supervisors, HIV testing counsellors	
FGD 4: Service providers (*n* = 6)	General Practitioners, Sexual Reproductive health educator, Peer educators, Outreach worker	
FGD 5: Transgender women (*n* = 6)	Transgender women, both HIV positive and negative, sex work	

Ethics approval was granted by the Johns Hopkins School of Public Health Institutional Review Board and the Myanmar Department of Medical Research, Ethical Review Committee.

## Results

In-depth interviews were conducted with 25 participants, 12 of whom identified as MSM (48%) and 13 as TW (52%). The median age of IDI participants was 23 years (range 18–42) and 16 (64%) were aged between 18 and 25. The majority of participants were single (*n* = 10), while 4 and 7 participants reported a current relationship with a casual and regular partner(s) respectively. Sixteen participants were HIV negative (64%), eight were HIV positive (32%) and one was HIV status unknown or undisclosed (4%). Five FGDs were also held with 35 participants; two with service providers (*n* = 8 and 6), one with MSM and TW community leaders (*n* = 9), one with hidden MSM community leaders (*n* = 6) and one with TW community leaders (*n* = 6) (Table 1).

### *“I HAVE NEVER ADMITTED I’M A MSM BECAUSE I AM HIDDEN”:* CHARACTERIZING THE LIVED EXPERIENCE OF HIDDEN MSM IN MYANMAR

Participant responses highlighted the perceived interconnectedness between sexual and gender identities in Myanmar, with participants expressing a belief that men who are sexually oriented towards other men share an inherent desire to dress and present in a feminine form:

When we become gays … also the hiddens who become gays... we want to look or behave like girls. Forget about the rules. As soon as we realize we are not straight, we want to look/behave like girls...

- Participant 1, FGD 2, Hidden MSM

Transgender women were therefore regarded as those who successfully embraced this desire while those who repudiated it were typically regarded as ‘hidden’ due to their efforts to maintain heteronormative behavior and presentation. As the following participant notes, expression of gender and sexual identity was often related to the external environment:

There are environments where hidden MSM cannot dress like *Apwints*… So they take hidden forms…

- Participant 3, FGD 2, Hidden MSM

These ‘hidden forms’ enabled MSM to conceal their sexual preferences by maintaining gender-confirming appearances and behaviors. As the above participant suggests, disclosure and concealment of sexuality was often contextual and tied to specific environments. This quote, and the one that follows, points to a fluidity of sexual and gender expression among MSM and TW in Myanmar:

*Interviewer:* First, have you heard of the words *Apone, Apwint,* etc?

*Participant:* Yes, I know

*Interviewer*: Which type would you identify yourself with?

*Participant*: I am more like *Apwint*

*Interviewer:* More *Apwint?*

*Participant:* Yes, because I am no longer *Apone.*

- IDI 34, TW, age 36.

A key influence on the disclosure of sexual and gender identity was the perception or expectation of stigma and discrimination. Participants directly connected the degree of anticipated or experienced stigma and discrimination to the extent to which sexuality was publicly disclosed or observed. For example, participants who were more ‘visible’ as sexual minorities, such as TW, or MSM who openly had relationships with male partners, more commonly experienced stigma from both significant others and the community. Consequently, concealment of their sexual identity and gender-conforming presentation enabled hidden MSM to avoid similar experiences to those described by this TW participant:

Of course, there are challenges. As I have a husband (long term male partner), I have to face more problems in my neighbourhood. I have to face things like that... Since I started identifying myself as a gay, there has been discrimination from my siblings and community.

- IDI 23, TW, age 22

The extent to which MSM felt the need to conform to traditional gender roles, including in the context of meeting family expectations to marry and have children, also had significant bearing on disclosure of sexuality. Underscoring the primacy of preserving good family relationships was an assumption that families would be disapproving of same-sex orientation. The following quote illustrates a possible consequence of disclosure to families:

To admit openly… that he is MSM, it will be difficult for him to be accepted by his family and community. There is a concern that the family will find out and abandon him.

- IDI 11, MSM, age 22

Participants engaged in a range of negotiated identity practices in order to conceal their sexuality from their family and community and avoid anticipated stigma and discrimination. Many described their general avoidance of other MSM and TW and their beliefs that any association would enable others to identify them as part of the MSM or TW community. While some avoided other MSM and TW all together, others restricted their socialization only to other hidden MSM:

I deny it when my family asked me. I have never admitted I’m a MSM either in the past and now because I am a hidden MSM and not an open type... I don’t know whether they pretend they don’t know about me. But they don’t like my *Apwint* friends and I choose *Apone* as my friends

- IDI 26, Hidden MSM, age 23

Other participants described a careful demarcation between their family and social lives, in order to maintain concealment of their sexuality around family, while selectively disclosing in certain social settings:

When I was in 8th or 9th Standard, I met some gays in the neighborhood. They told me “Hey ... Here. You have to dress like this.” and “A gay has to live like this.” …My family did not allow me to live in a gay (feminine) appearance. Even my hair was short like a boy and I wore a *longyi* (sarong typically worn by men in Myanmar). So, I had to meet them secretly at night.

- IDI 27, MSM, age 24

Selective disclosure also meant that participants' sexual identity and gender presentation was not static; many participants described a fluidity of sexual and gender performance which highlights the situational and contextual nature and application of sexual identity labels in Myanmar. This participant shares their experience of moving between transgender and male forms:

Although I was a gay, I still wanted to live like a boy. I mean – I wanted to dress like a boy. Others dressed like women at night. I did it sometimes. Also these days, as I am working as a dancer in a *Zat* band (traditional dance band), I dress like a woman. For the time being, as our team disbands for the season … I stay with friends, in places where there are MSM.

- IDI 27, MSM, 24 years old

### *“HIDDEN MSM DO NOT TRY TO LEARN MUCH [ABOUT HIV] THINKING PEOPLE MIGHT FIND OUT THEIR MSM STATUS”*: BARRIERS TO ACCESSING HIV PREVENTION SERVICES AMONG HIDDEN MSM

The behaviors and concerns of potential discrimination and stigma among hidden MSM described above presented a range of barriers to their engagement with HIV prevention services. Most notably, many participants described the perceived threat of disclosure of their sexual identity associated with HIV testing services – either related to the need to disclose or discuss potential sexual risk behaviours, or questions that may be raised following a possible HIV diagnosis:

With hidden MSM, most families don’t know them. Since they are MSM, it will be a bit more difficult for them to test (in case) somebody finds out while he is testing. (Also) If he is hidden and he has it [HIV] and people don’t know his orientation, I think it can hurt him mentally.

- IDI 26, Hidden MSM, age 23

The priority given to concealing sexuality by hidden MSM shaped their preferences for HIV testing locations and providers. Many described the paramount importance of maintaining privacy and confidentiality in relation to their sexual orientation. While many MSM and TW participants expressed a preference for tailored HIV services, hidden MSM tended to weigh the perceived suitability of such MSM and TW-specific services against the threats to their confidentiality that these services were seen to pose. This ambivalence among hidden MSM towards MSM- and TW specific services was compounded by their avoidance of other MSM and TW. This participant describes an example of such avoidance from a fellow MSM waiting for HIV testing:

When I took a blood test in June, there was another *Apone* and I recognized him as an *Apone* at a glance. He was using the umbrella with anti-discrimination day label. I knew he was *Apone*. And he most probably recognized me as well. But he didn’t want me to know he came there for a blood test. He was sitting in a corner silently. He didn’t want me to notice him.

- IDI 24, Hidden MSM, aged 30

Government-provided services were viewed unfavourably by most MSM and TW, often related to past experiences or expectations of stigmatization and discrimination by government staff. This was particularly felt by TW participants and more ‘open’ MSM:

With the NGOs, since we are MSM, they do counselling regarding sexual disease if we have it. They treated us warmly without discrimination. That is the difference. It is difficult for MSM to enter government clinic. They don’t go there. There are many MSM staffs at NGOs. We just go where we are comfortable.

- IDI 33, TW, age 22

While both ‘open’ MSM and TW, and hidden MSM shared this fear of stigmatization by health staff, for hidden MSM, the specific requirements of government services, as described below, and the inherent risk of indiscretion that these requirements carried presented additional barriers to accessing HIV testing at government services:

If an *Apone* like me went to NAP [National AIDS Programme], there would be a lot of challenges. They insist we bring one family member to their counseling session. And they don’t do history taking and counseling sessions individually. They ask about sexual issues in front of several doctors and female nurses. And they tell us to bring our male partners for blood test.

- IDI 24, Hidden MSM, age 30

In general, health-seeking behavior among hidden MSM was largely influenced by the extent to which services could offer anonymity. Below describes a participant’s recent visit to a HIV testing clinic that served MSM and TW clients as well as the broader community. Service satisfaction was related to the relative anonymity provided by this clinic, coupled with the skill and sensitivity of the service provider towards MSM and TW clients:

Although it was a general clinic, many MSM went there. It was good for me as there were not too many MSMs. Hidden ones can also visit. All kinds of them. It was good because there was an MSM project (also at the clinic) ... I felt more secure and safe. It is better for the clients.

- IDI 24, Hidden MSM, aged 30

The need for anonymity also held important implications for hidden MSM diagnosed with HIV. As this participant describes, hidden MSM may choose to prioritize the concealment of their sexuality over their own health and well-being. For some, a HIV diagnosis would be regarded as an unwelcome intrusion of their private ‘hidden’ life, into their daily, more ‘visible’ lives, such as the need to take daily treatment for example.

I think *Apones* will face more challenges (with HIV testing). They are hiding themselves and when they get a positive result, they can have more worries and emotional problems. Some don’t want to disclose even if they get a positive result. And you know? They might think “Why should I care about a positive result? I am not going to seek medical care.” *Apwints* have more guts to disclose and discuss. They will seek medical care if they get a positive result. I think they have more knowledge than *Apones* do; compared to *Apones*, many *Apwints* know about HIV. *Apones* do not try to learn much (about HIV) thinking people might find out their MSM status.

- IDI 16, MSM, age 23

The need to navigate between these two worlds likely impacts the perceived suitability and appropriateness of HIV treatment and support. The following participant describes the differentiated perspectives of home-based peer-support - a service commonly provided by NGOs to promote adherence to HIV treatment and ensure ongoing psychosocial wellbeing - among hidden MSM and TW:

Interviewer: Was it okay when there were visitors at home?

Participant: For transgender, they mostly spend time outside the home. And those who are accepted by their families, home visits are okay. For hidden MSMs, home visits are not okay.

Interviewer: So, you make appointments and meet him/her in a convenient place?

Participant: They (hidden MSM) just don’t want people around.

- IDI 24, Hidden MSM, age 30

### *“WE ARE PEERS AND WE KNOW WHAT HIDDEN MSM DO BUT, AT THE COUNSELING SESSION, THEY WOULD SAY THEY ARE NOT THAT KIND OF PERSON … WHAT DO WE DO THEN?”* SERVICE PROVIDER AND COMMUNITY LEADER PERCEPTIONS OF BARRIERS TO ENGAGING HIDDEN MSM IN HIV PREVENTION

Data from service providers and community leaders highlighted the ways in which characteristics of hidden MSM presented barriers to effectively engaging this group in HIV prevention. In discussing the challenges, service providers demonstrated an awareness of concerns and inner struggles faced by hidden MSM and recognized the impact this had on their approaches to service provision, as this service provider demonstrates:

Interviewer: How do you identify open and hidden type?

Participant: Hidden type hides himself. It is quite difficult to persuade him to do blood test. The thought is that what if someone sees him if he comes here?

- Participant, FGD 3, Service Provider

Some service providers observed that hidden MSM had low levels of HIV prevention knowledge and were less likely to engage in protective behaviors compared to other MSM and TW:

Hidden MSM don’t admit it (that they have sex with men). They don’t always use condoms. Even if they use condoms, they sometimes don’t know if they are wearing the condoms correctly or inside-out.

- Participant, FGD 1, MSM Community leader

This behaviour may be a result of a deliberate avoidance of the purchase of or access to condoms in order to conceal sexual behaviours, or reflect the limited knowledge of HIV prevention and safe sex practices due to minimal engagement with HIV prevention services and providers. For example, this service provider describes their experience encountering MSM who deny their sexual orientation and therefore their need for HIV-related health promotion during community-based outreach and health promotion:

In hidden types, they don’t even want to be known among themselves. They live with their pride. When you look at them, they look like real men… When you tell them to do blood test, they are scared to death. When you tell them about health education, they told (us) “We are not gays. Since I don’t think of myself as gay, don’t tell me those things.”

- Participant, FGD 3, Service provider

Some participants believed that the constant need among hidden MSM to hide their same-sex preferences and behaviors resulted in negative psychological effects such as feelings of internalized stigma and homophobia. In some cases, these negative feelings led to a sense of antipathy towards other MSM and TW. This hidden MSM reflects on these feelings arising from self-stigma and an ongoing need to conceal their sexual identity:

They (hidden MSM) can’t let the community know at all. And they can’t let their families know at all. So, finally they develop an attitude that they can’t even accept themselves as MSM….They have a gay instinct but they hide themselves so much and try so hard to prove they are not gays .. and then they gradually begin to hate gays.

- Participant 5, FGD 2, Hidden MSM community leader

The denial of same-sex behavior by hidden MSM was seen as a strategy to distance themselves from other MSM and TW as part of their broader attempts to conceal their sexuality. This behavior persisted even in situations where MSM sought HIV testing. Service providers noted how the reluctance of hidden MSM to discuss same-sex behaviors limited the ability of HIV testing staff to accurately assess risk and provide appropriate counseling. As this service provider notes, prevarication from hidden clients during HIV testing was a common experience:

We are peers and we know what hidden MSM do. But, at the counseling session, they would say they are not that kind of person … What do we do then? As we are peers, we know who is doing what. And things were okay when they first talked to us... Then, when they faced the counselor, and when the counselor started explaining, they said “I’m not that kind of person” and come out of the counseling room.

- Participant, FGD 1, MSM Community leader

Service providers also acknowledged the negative ways non-disclosure of sexuality intersected with HIV diagnosis and the impact this had on HIV treatment. As this following quote highlights, the need to keep sexual and family lives separate prevented some HIV-positive MSM from accessing family support. This quote also illustrates the contextualized nature of disclosure of sexuality among MSM as well as the potential impact - such as delayed or disrupted treatment - on access to HIV prevention and care services:

Interviewer: Are there conditions where MSM cannot come for HIV treatment all?

Participant: I have seen one… He is only 22. He lives with his aunt. She discriminates him because he is MSM. He came to our clinic and said he wants to test. He is “out of the closet” type but he lives like “in the closet” type at home in front of his aunt. When tested, his HIV test was positive. When I showed him the long-term plan (for treatment and care) he said that his aunt does not agree with him being an MSM (so) he has to live like a man. She does not like him going out…. The aunt will be worse if he has HIV because she doesn’t like him being MSM.

- Participant, FGD 3, Service provider

In response to these complexities, service providers described the different approaches they adopt to engage hidden MSM, demonstrating an understanding of the characteristics of this group and a level of empathy for their struggles. In particular, many service providers were cognizant of hidden MSM’s need for privacy and discretion. This service provider reflects on how these priorities are reflected in their professional practice:

The one who is doing prevention knows them (hidden MSM). However, they hide. I know I cannot approach them like they are gay. I call them big brother or little brother. They liked to be called like that.

- Participant, FGD 3, Service provider

Conversely, while many service providers and community leaders expressed understanding and empathy towards hidden MSM, others expressed feelings of frustration and impatience in the face of the difficulties they experienced working with this group. Underpinning this frustration was an expectation of responsibility among MSM and TW to prevent the spread of HIV. The following quote assumes a sense of community that underpins this sense of responsibility, however this sentiment is unlikely to extend to hidden MSM. This quote also speaks to the importance of rapport- and trust-building in the HIV prevention work carried out by peers - which is often developed over time and through repeated interactions - and is typically a precursor to longer-term engagement of MSM and TW with HIV prevention services. This community leader reflects on the challenges to engaging with hidden MSM and conveys a sense of frustration:

We want to help them understand such things as HIV prevention. It’s not that we have left the hidden ones out but they have left us. There will be no discrimination if they have open discussions with us… They need to cooperate by doing their part…. They are not interested in how to use condoms to prevent HIV and how to take treatment. We don’t leave them out but they have left us.

- Participant, FGD 1, MSM Community leader

Responses from some service providers and community leaders also conveyed a sense of powerlessness against the numerous influences that prevented MSM from more freely disclosing their sexual preferences and behaviors. These responses also displayed their recognition of the need for supportive and enabling environments if such behaviors were to change:

What can we do when hidden people from the community don’t disclose? … Rather than tell them to take HIV tests, they don’t even know what HIV is and what AIDS is. To face this thing (HIV testing) is something which comes later. From the very beginning, their parents did not accept them being gays. If these problems (like a positive HIV diagnosis) are added, a lot of them will be dead.

- Participant, FGD 1, MSM Community Leader

This quote also hints at recognition by some community leaders and service providers of a hierarchy of priorities for hidden MSM that may supersede the need for HIV testing and prevention. For example, denying or concealing same-sex orientation may enable hidden MSM to maintain family relationships and support, which may be disrupted by an HIV diagnosis that may force hidden MSM to reveal their sexuality and potentially upset family dynamics.

## Discussion

Increasing the reach of HIV prevention programs among hidden MSM has been identified as a national priority for HIV control efforts in Myanmar [27], yet much remains unknown about this group. This study furthers our understanding of the characteristics of hidden MSM and how they influence levels of engagement with HIV prevention services. This study identified a number of barriers to HIV service access and uptake which largely stemmed from hidden MSM’s preference to conceal same-sex behaviour and orientation; for example, through avoidance of other MSM and TW, reluctance to attend HIV prevention services, denial of same-sex behaviors and adopting gender-conforming behaviors and presentations, particularly around family. Additionally, fear of stigma and discrimination and a need for privacy and confidentiality were key factors in shaping preferences for HIV prevention services among hidden MSM. Service providers and community leaders corroborated the experiences and characterizations of hidden MSM and noted the unique ways in which these barriers impacted their ability to engage hidden MSM in HIV prevention. Taken together, these findings provide some context for the low rates of HIV testing currently observed among MSM and TW in Myanmar [4] and carry important implications for the enhancing service demand through the development of culturally-relevant HIV prevention programs that are acceptable and can adequately address the specific needs and concerns of hidden MSM in Myanmar.

In this study, we found that non-disclosure of same-sex behaviour and preferences was commonly employed by MSM as a strategy to avoid stigma and discrimination from their families and communities. In Myanmar, although rarely enforced, homosexual sex remains constitutionally illegal and contributes to an environment in which sexual minorities continue to report ongoing harassment and discrimination [15]. The criminalization of homosexual sex has also been associated with reduced levels of access to HIV services among MSM and TW [28]. Service providers in Myanmar have noted how the current legal environment complicates the delivery of HIV prevention services by preventing community-based organizations from official state registration [18]. Anti-homosexual legislation works to legitimize discrimination and negatively impacts the acceptance of sexual minorities, promoting concealment of sexual identity and behaviors among MSM. Across Asia, non-disclosure of sexuality has been associated with higher rates of condomless sex [29] and lower rates of HIV testing among MSM [30, 31]. Similarly, in this study, we found that the requirement to disclose same-sex behaviors in order to receive appropriate HIV services and commodities effectively inhibited hidden MSM’s access to these services; this reluctance hinders the provision of appropriate and tailored care, including specific risk-reduction counseling. Current WHO guidelines for example, recommend the provision of condoms and lubricant and the opportunity for anal cancer screening for all MSM [32], however these services are unlikely to be offered if same-sex behaviors are not revealed.

This study found that many hidden MSM engage in negotiated identity practices in order to carefully manage the concealment and disclosure of their sexual identity. Often this behaviour was tied to environment, with many hidden MSM describing adoption of gender-conforming behaviors and presentation around family and the careful demarcation of social/sexual lives and family/community contexts. This demarcation often involved the public avoidance of other MSM and TW and underscores the importance of privacy and confidentiality as a key determinant of health seeking behaviours and preferences for HIV prevention services among hidden MSM.

Peer-based outreach and health promotion is a fundamental component of Myanmar’s national response to HIV [27] and has been identified as a key strategy to drive up rates of HIV testing among MSM [4]. Yet the demonstrated reluctance among hidden MSM to publically engage with peer outreach workers may undermine the potential effectiveness of national HIV prevention strategies and programs in Myanmar. While some peer workers adopted inconspicuous approaches to engaging hidden MSM in outreach, and in doing so, demonstrated an awareness of this group’s preference for privacy (for example, using more generic terms to address hidden MSM such as “*friend*” or “*brother*”), others indicated a sense of frustration at the difficulty of engaging hidden MSM that suggested limited empathy for the psychosocial struggles they experienced. There is an opportunity to strengthen community-based HIV prevention programs by ensuring all peer staff are comprehensively trained to both recognize and deal with these psychosocial struggles, including internal stigma and homonegativity, experienced specifically by men unwilling or unable to disclose same-sex preferences.

In general, responses to HIV are often based on easily identifiable risk categories, including “MSM” and “TW”, which many have argued detract from properly appreciating the complexity of gender and sexual expression and the way this shapes HIV vulnerability and risk [33–35], including in the Asian context [36–38]. Yet, the reappropriation of the term ‘MSM’ as an identity category has been noted, signalling the transformation of ‘MSM’ from epidemiological term to subject position [39]. In this study, numerous participants used the term’ MSM’ as an identity label, parallel to local parlance as has been noted in other settings among same-sex attracted men [40]. Neither continued reliance on the binary MSM/TW categories alone, nor the wholesale integration of local terminology in HIV programing would be sufficient; as argued by Thomann [40], the key to more responsive HIV programing is the recognition of the diverse lived experiences contained within local identities and how this intersects with other local and social realities to shape vulnerability to HIV. Indeed, the differing experiences and perspectives among the various sexual and gender identities, even within the same identity group, documented in this study highlights the importance of service provision that can appropriately cater for different groups and their heterogenous needs. In Laos, for example, behaviorally bisexual men demonstrated reluctance to attend MSM- specific services [23], underscoring the importance of identifying as the intended recipient of HIV services. In this study, attendance at MSM-specific services was believed to present a high risk of association with the MSM community. Yet, while general testing clinics were viewed as providing greater anonymity, these clinics also presented barriers for hidden and disclosing MSM and TW including experiences of stigmatization and discrimination from health care staff. Owing to similar concerns for confidentiality, recent research found that hidden MSM were more likely to seek treatment from public clinics, general practitioners or self-medicate compared to other MSM [18] or may simply eschew HIV services all together [20]. In such contexts, generic Men’s Clinics staffed by MSM-friendly providers who can discretely provide services appropriate for this group’s sexual risk behaviors may be an appealing option for hidden MSM. The latest National Strategic plans identifies ‘One stop shops’ as a potential strategy to increase HIV testing rates among MSM [4]; the findings from this study suggest that such an approach would be well-received by this group, provided staff were appropriately trained and aware of specific barriers encountered by hidden MSM. Delivery of comprehensive training for staff in mainstream HIV services and other government health services, and inclusion into medical and nursing curricula, to enhance both sensitivity and capacity to provide appropriate and tailored services to sexual minorities would also increase the ability of providers in these settings to more comprehensively address the specific needs of MSM.

Myanmar is currently experiencing a period of significant transition and development, which has included an evolving interpretation of homosexuality (including the adoption of more ‘Western’ terminology such as ‘homo’, ‘gay’ and ‘queer’, particularly among younger, urban individuals and/or those connected to the NGO sector as demonstrated by some study participants; [14, 18, 41]). This ongoing development has also led to changing social and political environments, characterized by an emergent LGBTI community and an increasing number of civil society groups and social networks for sexual minorities, many of which are actively engaging in policy discussions and highlighting issues in their communities [18]. In 2012, Myanmar held its first gay pride parade [42], a sign of the shifting attitudes and growing tolerance towards sexual minorities in Myanmar. However the prevailing legal environment sustains a sense of fear of discrimination and possible arrest among some MSM and TW whose desire to avoid both may supersede the importance of HIV testing and engagement with HIV prevention services, particularly those that pose a risk of disclosure, and should be recognized as a key barrier to service access. The expanding availability of information and communication technology (ICT) and infrastructure [43] in Myanmar, including a growing ubiquity of cyber networks among MSM communities [44], offers opportunities to engage MSM and TW in HIV prevention who may otherwise be reluctant to do so. ICT-based approaches have demonstrated some positive results in engaging MSM in HIV testing [45–48], including among non-gay identified MSM [49], and are increasingly used to engage hard-to-reach and hidden MSM populations across Asia [50, 51]. The evolving social and political context offers opportunities to reduce some of the identified barriers to engaging MSM through increasing the social acceptance of sexual minorities in Myanmar, while technological advances can be used to enhance the reach of hidden MSM in HIV prevention through the innovative use of online and new technologies. Together, this new landscape can promote an enabling environment in which some of the barriers to engaging hidden MSM in HIV prevention services described in this study are reduced.

This study has several limitations that should be considered alongside the results. Study participants were recruited from partner NGO organizations and their views and perspectives may not represent MSM and TW who are harder to reach or not currently connected to services. Additionally, this study did not specifically ask participants to identify their sexual identity a priori using the locally accepted terms described in this paper and we cite them insofar as they were used by participants themselves during FGDs and IDIs. Therefore, it is possible that some of the references to hidden MSM may be in relation to *Thange* participants, who are also considered ‘hidden’ but who are likely to face unique barriers related to their specific sexual identity. More research is warranted to explore how the barriers faced by hidden MSM described in this study may be uniquely and variedly experienced by *Thange* and *Apone* MSM respectively. Lastly, our sample was recruited from Yangon, the largest city in Myanmar and may not reflect the lived experiences of MSM and TW in rural or remote areas where access to HIV services and tolerance towards gender non-conformity may be lower. However, we believe the findings are relevant to other urban locations in Myanmar which are typically characterized by larger populations of MSM and TW compared to peri-urban and rural areas [4].

## Conclusion

As highlighted by this study, MSM in Myanmar who are unwilling or unable to disclose their sexual behavior and preferences face multiple and overlapping barriers to accessing HIV testing and other prevention services. Strategies such as building the capacity of peer service providers to identify and respond to psychosocial challenges experienced by non-disclosing MSM, sensitivity training to reduce discriminatory attitudes and practices among mainstream health providers and government workers, and innovative approaches such as one stop shop services and ICT-based health promotion that can discretely provide services tailored to the distinct needs of MSM and TW, while ensuring their privacy, may help circumvent some of the barriers identified in this study. Such activities should be considered as part of an overarching HIV prevention strategy that also addresses the broader structural barriers related to stigma and discrimination in the general community to improve the social acceptability of sexual minorities and create an environment in which hidden MSM can comfortably discuss same-sex behaviour and access services appropriate for their risk behaviors.

## Abbreviations

FGD: Focus group discussion
ICT: Information and communication technology
IDI: In-depth interviews
MSM: Men who have sex with men
NGO: Non-governmental organisation
TW: Transgender women
